# Transformation of silver nanoparticles released from skin cream and mouth spray in artificial sweat and saliva solutions: particle size, dissolution, and surface area

**DOI:** 10.1007/s11356-020-11241-w

**Published:** 2020-10-23

**Authors:** Jonas Hedberg, Madeleine Eriksson, Amina Kesraoui, Alexander Norén, Inger Odnevall Wallinder

**Affiliations:** KTH Royal Institute of Technology, School of Engineering Sciences in Chemistry, Biotechnology and Health, Department of Chemistry, Division of Surface and Corrosion Science, Stockholm, Sweden

**Keywords:** Silver nanoparticles, Consumer products, Dissolution, Sweat, Saliva, Particle size

## Abstract

The use of silver nanoparticles (Ag NPs) in consumer products can result in diffuse environmental dispersion of both NPs and ionic silver. This study investigated the transformation of Ag NPs present in two consumer products (skin cream, mouth spray) in terms of release of Ag NPs and ionic silver and changes in particle size in artificial sweat and saliva solutions. Large differences in silver release were observed with the smaller sized Ag NPs in mouth spray releasing more silver compared with the Ag NPs of the skin cream. Substantial particle agglomeration took place in both artificial sweat and saliva, forming large-sized agglomerates (> 100 nm). The amount of dissolved silver in solution after 24 h was less than 10% of the total amount of Ag NPs for both products. The results show that the Ag NPs of these consumer products will largely remain as NPs even after 24 h of skin or saliva contact. The use of normalization by geometric surface area of the particles was tested as a way to compare dissolution for Ag NPs of different characteristics, including pristine, bare, as well as PVP-capped Ag NPs. Normalization of silver dissolution with the geometric surface area was shown promising, but more extensive studies are required to unambiguously conclude whether it is a way forward to enable grouping of the dissolution behavior of Ag NPs released from consumer products.

**Electronic supplementary material:**

The online version of this article (10.1007/s11356-020-11241-w) contains supplementary material, which is available to authorized users.

## Introduction

Silver nanoparticles (Ag NPs) are used in a large variety of consumer products (Hansen et al. 2016; Vance et al. 2015), mainly to provide antimicrobial effects (Zhang et al. 2016). Silver can be released as Ag NPs and/or ions from such consumer products and interact with humans and the environment. This release has spurred numerous investigations on transformations of Ag NPs upon dispersion, including aspects such as surface chemistry, dissolution, and toxicity (Cronholm et al. 2013; Hedberg et al. 2019a; Levard et al. 2013a; Levard et al. 2012; Levard et al. 2013b; Zhang et al. 2016). The toxic potency and the physico-chemical properties of the Ag NPs govern possible hazards on human health and the environment, combined with the actual dose (concentration) of dispersed Ag NPs (Arvidsson et al. 2011; Benn and Westerhoff 2008; Coll et al. 2016; Gunawan et al. 2017; Zhang et al. 2016).

The regulatory framework is trying to catch up with the rapid development and use of new nanomaterials (NM), including Ag NPs, to ensure their safe and sustainable use and handling. In 2018 the Swedish Chemicals Agency (KemI), for example, implemented a rule that producers of NM-containing products must register properties (e.g., size and charge) of added NMs (Kemikalieinspektionen 2015). However, this database and other collections of data on NMs largely lack information on actual release rates of Ag NPs from such products (Hansen et al. 2016), as well as on properties of released NMs (size, composition, morphology, etc.) (Koivisto et al. 2017). This shortage of data is also evident from the scientific literature for which most studies that addresses the release of NMs from consumer products (67% as of 2016) do not include any information on NM transformation products (Caballero-Guzman and Nowack 2016). The need for more real-world realistic test systems has moreover been recognized (Mitrano et al. 2015b; Mitrano and Nowack 2017).

Ag NP–containing consumer products are among the most studied NM-containing products. Available studies include transformation information of Ag NPs during simulated exposure scenarios of, e.g., socks (Benn and Westerhoff 2008), toothbrushes (Mackevica et al. 2017), food containers (Mackevica et al. 2016), dietary supplements (Radwan et al. 2019), surface sanitizers (Radwan et al. 2019), mouth sprays (Quadros and Marr 2011), fabrics (Kulthong et al. 2010), and various products for children (Quadros et al. 2013; Tulve et al. 2015). Pristine Ag NPs as model systems for Ag NPs released from consumer products have been studied to understand possible transformations and environmental interactions. These investigations include, for example, interactions with laundry cycle component and surface water (Hedberg et al. 2014b), as well as the influence of light on Ag NP aging (Mitrano et al. 2015a). Some studies report extensive agglomeration of Ag NP in artificial saliva (Lin-Vien et al. 1991), whereas others report reduced agglomeration in authentic saliva due to the presence of organic compounds (Ngamchuea et al. 2018). The presence of capping agents/adsorbed ligands on Ag NPs has shown to influence their stability, as illustrated by, for example, a reduced extent of particle agglomeration and altered chemical stability. These differences in stability can influence dissolution rates and kinetics compared with bare particles (Liu et al. 2018; Molleman and Hiemstra 2017; Radwan et al. 2019). A study that compared Ag NPs in socks with pristine particles showed that a 24 h incubation of the pristine NPs in wastewater could serve as a model for the behavior of transformed NPs from a consumer product (Mohan et al. 2019). However, as there are several hundreds of different Ag NP–containing consumer products but scarce data on the transformation and characteristics of released Ag NPs, it would be very valuable to simplify and group these products in terms of, for example, their dissolution characteristics. This would speed up investigations on the release and transformation of Ag NPs (e.g., size, dissolution, surface properties) from different consumer products on the market that can be used in modeling of hazards and environmental fate (Caballero-Guzman and Nowack 2016; Mitrano et al. 2015b; Mitrano and Nowack 2017).

This work studies transformation of Ag NPs released from two different consumer products (skin cream, mouth spray) in synthetic sweat and saliva. Ag NPs are added to these kind of products as an antimicrobial agent (Khaksar et al. 2019a), and other personal care products incorporating Ag NPs include, for example, shampoo and toothpaste (Benn et al. 2010) Ag NPs represent a smaller part of the NPs in personal care products, with TiO_2_ and ZnO being the most commonly used NPs (Keller et al. 2014). The release of Ag to graywater (household water) has been identified as an important route for dispersion of Ag emissions from Ag NPs in personal care products (Khaksar et al. 2019a).

The investigations include quantification of released Ag NPs and their extent of dissolution into silver ionic species, as well as measurements of surface composition and changes in particle size distribution over a 24 h time period. The behavior of the Ag NP–containing consumer products and pristine Ag NPs are compared in terms of dissolution normalized by the geometric surface area (based on the primary size of the NPs at dry unexposed conditions). The aim is to contribute with knowledge that elucidates the applicability of using pristine Ag NPs, or reported findings for Ag NPs in consumer products, as models to assess the release of ionic silver from Ag NPs in consumer products. Normalization by geometric surface area is clearly a simplified approach since other properties certainly will influence the dissolution process, such as differences in particle size and presence of capping agents (Hedberg et al. 2019b). The geometrical surface area is on the other hand a parameter which in most cases is readily available, as opposed to, for example, the fractal dimension of agglomerates, effective surface area of particles/agglomerates in solution, or their surface composition in solution. This study will investigate whether there is a trend between surface area and dissolution and to assess differences between different kinds of Ag NPs. Literature findings on dissolution is compiled together with data generated in this study for Ag NP–containing consumer products and pristine Ag NPs (bare, PVP-capped). Investigations of both capped and bare Ag NPs serve the purpose to represent Ag NPs with significantly different initial surface properties at the starting point of the exposure in the synthetic body fluids of interest.

## Materials and methods

### Solutions and chemicals

Table 1 gives the compositions of artificial sweat (ASW) and artificial saliva (AS), using ultrapure water (18.2 MΩ cm resistivity, Millipore, Solna, Sweden) as solvent.

**Table 1:** Chemical composition of artificial sweat and artificial saliva

	Artificial sweat (CEN 2011)	Artificial saliva (Oh and Kim 2005)
pH	6.5	6.75
NaCl (g/L)	5.0	0.4
Lactic acid (g/L)	1.0	–
KCl (g/L)	–	0.4
Urea (g/L)	1.0	1.0
CaCl_2_·H_2_O (g/L)	–	0.795
NaH_2_PO_4_·H_2_O (g/L)	–	0.78
Na_2_S·9H_2_O (g/L)	–	0.005

All chemicals were of analytical grade (p.a.) or puriss. The glassware was immersed in 10% HNO_3_ for at least 24 h and thoroughly rinsed with ultrapure water prior to usage.

### Silver nanoparticles

The consumer products were manufactured by MaxLab (Serbia) and marketed under the name “Silversalva” (skin cream containing Ag NPs) and “Silversept munsprej” (mouth spray containing Ag NPs). According to the manufacturer information, the skin cream and the mouth spray contain 30 and 20 mg Ag/kg product, respectively. Other ingredients of the skin cream include calendula, allantoin, vitamin B5, and glutamine. Ingredients of the mouth spray were *Acacia senegal* (a herbal constituent), panthenol, *Mentha x piperita* oil, *Salvia officinalis* oil, *Pimpinella anisum* seed oil, *Thymus zygis*, and herb oil. Pristine PVP-capped (40 kDa) Ag NPs were purchased from Nanocomposix (San Diego, USA) with a primary size of 50 nm according to the manufacturer. The bare Ag NPs were purchased from American Elements (Cleveland, USA) in a purity of 99.9%.

### Electron microscopy

Transmission electron microscopy (TEM) imaging of bare and PVP-capped Ag NPs was performed using a Hitachi HT7700 instrument, operating at 100 kV. The bare Ag NPs were prepared by dispersing and sonicating (see details elsewhere (Pradhan et al. 2016)) the particles in butyl alcohol at a concentration of 1 g/L for 15 min. The suspension was then pipetted onto TEM copper grids coated with Formvar® films (Ted Pella, USA) from which the solvent evaporated under ambient laboratory conditions. The PVP-capped Ag NPs were prepared by placing a drop from the stock solution (5 g/L Ag NPs) onto the TEM grid. Excess solution was removed using a paper tissue. All TEM images were recorded in bright field mode except for the Ag NPs in the skin cream (dark field mode).

The Ag NPs in the skin cream and the mouth spray were imaged using a JEOL 200 kV 2100F field emission microscope operated in scanning beam mode (STEM). This was combined with energy-dispersive spectroscopy (EDS) microanalysis, using a windowless silicon drift detector X-MaxN TLE from Oxford Instruments and the Aztec Software, to identify the Ag NPs. Samples of the skin cream were prepared by heating the skin cream at 500 °C for 30 min in a muffle furnace to reduce its organic matter content. The ash was dispersed in butyl alcohol in a sonication bath for 20 min before being deposited onto the copper grid followed by solvent evaporation at ambient conditions. The mouth spray was prepared placing a drop of the stock solution on the grid removing excess liquid using a blotting paper.

### Dissolution studies

The Ag NPs were added to the different test solutions in Nalgene© jars that were incubated for 20 min, 1 h, and 24 h using a Stuart S180 Platform-rocker incubator (bilinear shaking, 22 cycles per min, 12° inclination). The exposures were conducted at 30 °C in ASW and at 37 °C in AS. All Ag NPs were investigated at a particle concentration of 2 mg/L in a total solution volume of 20 mL. The PVP-capped Ag NPs were diluted directly from the stock suspension.

The bare Ag NPs, initially in dry powder form, were sonicated into a stock solution of 1 g Ag NPs/L. Details of the sonication settings and procedure are given elsewhere (Pradhan et al. 2016). In short, the delivered acoustic energy was 1.2·10^6^ J/L by means of a Branson Sonifier using a micro tip for 15 min. The solution was cooled in an ice bath during the sonication process (Pradhan et al. 2016). The sonicated stock suspension was further diluted to a particle concentration of 2 mg Ag NPs/L. Both the mouth spray and the skin cream were diluted directly from the product into the Nalgene jars. Three independent replicas were investigated and analyzed for all dissolution studies in ASW and AS.

### Quantification of released Ag from the Ag NPs

From each exposed sample and time point, solution samples were collected and separated into a filtered and a non-filtered sample. Membrane filtration was performed to determine the fraction of released silver ions in solution. The remaining concentrations of Ag NPs in solution after the exposures and time periods were calculated by subtracting this fraction from the total amount of silver in the non-filtered samples. Filtration was made by passing 6 mL of the sample solution through an alumina filter (20 nm pore size, Anotop filter, Whatman) after which 15 μL of 65% HNO_3_ was added to preserve the samples (pH < 2) and to dissolve the Ag NPs prior to analyses of the total silver concentration.

Atomic absorption spectroscopy (AAS) was employed to determine the silver ion concentration in solution using a Perkin Elmer AAnalyst 700 instrument in graphite furnace mode. Calibration standards of 7.5, 15, 30, and 45 μg Ag/L were prepared from a 1 g Ag/L certified standard (Perkin Elmer). Recovery measurements of added Ag ions (of similar concentrations as the calibration standards) in AS and ASW resulted in a 80–90% recovery. The detection limit, 1.5 μg Ag/L, was estimated from three times the standard deviation of the blank samples. Calibration standards were analyzed every 5th sample, and re-calibration was performed if the calibration standard deviated more than 10% from the stipulated value.

### Particle size

The hydrodynamic particle number distribution was investigated by means of nanoparticle tracking analysis (NTA, Malvern Nanosight NS300, Uppsala, Sweden) using a 405-nm laser and an acquisition time of 60 s, repeated three times. Three independent samples were investigated for each time point. The camera level was intentionally kept very low (camera level 4) in order to target the Ag NPs, thereby excluding information from particles of organic matter present in the mouth spray and the skin cream during the measurements (Mehrabi et al. 2017).

### Surface characterization

A Horiba Yvon Jobin HR800 Raman spectrometer with a laser wavelength of 532 nm and a 50X objective was used for surface characterization of the Ag NPs in the mouth spray, the PVP-capped Ag NPs, and the bare Ag NPs. No measurements were possible for Ag NPs of the skin cream. Three different spots were investigated for each particle type with the laser beam softly focused to avoid any beam damage. The samples were checked by means of optical microscopy before and after the measurements to assure no laser-induced damage. For the mouth spray, a drop of the spray was deposited onto a glass slide and left to evaporate before the Raman investigation. The measurements were focused on agglomerates of Ag NPs that could be identified through the optical microscope (see Fig. S1 in supporting information) and that resulted in a very large signal owing to the surface-enhanced Raman effect (Moskovits 2005). The PVP-capped Ag NPs were investigated in the stock solution as a drop on a glass slide. The Raman investigations of the bare Ag NPs were conducted on the dry powder.

### Zeta potential

A Zetasizer Nano ZS instrument (Malvern Instruments, UK) was used to estimate the zeta potential of the NPs in ASW and in AS. The measurements were conducted at 25 °C in triplicate readings. The Smoluchowski approximation was used for modeling of the zeta potential distribution. The PVP-capped Ag NPs and the mouth spray Ag NPs were diluted in 10 mM NaCl to a concentration of 0.1 g/L Ag NPs. The measurements were performed in 10 mM NaCl as the high ionic strength of AS and ASW make zeta potential determinations difficult due to screening of surface charges (Skoglund et al. 2017). The bare Ag NPs were sonicated prior to the measurements; see details elsewhere for sonication settings (Pradhan et al. 2016).

### Chemical equilibrium calculations

Chemical equilibrium calculations of released silver in solution were performed using the Medusa software (Puigdomenech 2001), with the chemical composition of the AS and ASW solutions as input values (detailed in Table 1).

## Results and discussion

### Properties of Ag NPs before exposure

**Fig. 1:**
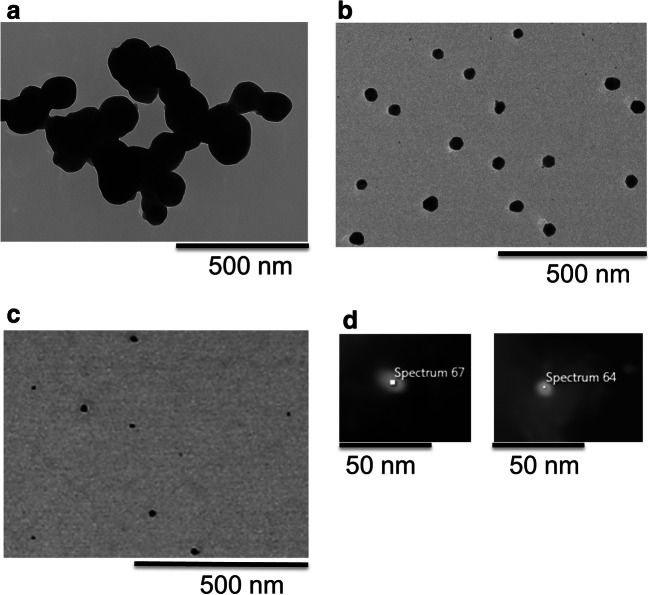
TEM images of bare Ag NPs (**a**), PVP-capped Ag NPs (**b**), Ag NPs in mouth spray (**c**), and Ag NPs in skin cream (**d**). All images were collected in bright field mode except for the skin cream obtained using dark field mode. EDS measurements confirmed the silver content of the text-marked spots in **d**. EDS spectra given in Fig. S2 for the Ag NPs in the mouth spray

**Fig. 2:**
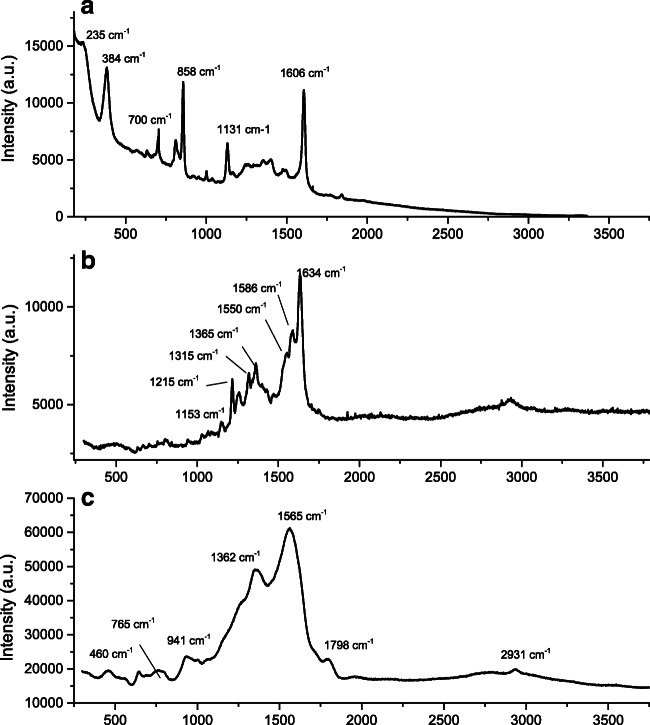
Raman spectra of Ag NPs. **a** bare Ag NPs. **b** Ag NPs in mouth spray. **c** PVP-capped Ag NPs

TEM images of the studied Ag NPs and their properties in terms of primary size (from TEM), zeta potential are presented in Fig. 1 and Table 2. Figure 2 shows the results of Raman spectroscopy.

**Table 2:** Primary particle size (based on TEM measurements), zeta potential, and surface compounds (from Raman measurements) of the investigated Ag NPs

Ag NPs	Primary size (nm)	Zeta potential (mV) in 10 mM NaCl	Surface compounds
Bare	50–150	0	Ag-O, Ag carbonate
PVP-capped	50–70	−46 ± 2	PVP
Mouth spray	2–20	−24 ± 3	Organic compounds
Skin cream	15–25	N/A	N/A

The TEM images of the mouth spray showed Ag NPs sized 2–20 nm, particles that in some cases formed agglomerates (see SEM picture in supporting information Fig. S3, S4). The size of the bare and PVP-capped Ag NPs was larger than those in the mouth spray, with primary sizes between 50 and 150 nm and 50–70 nm, respectively. The Ag NPs in the skin cream were in the size range of 15–25 nm.

The zeta potential measurements in 10 mM NaCl showed the bare Ag NPs to be uncharged (0 mV), and both the PVP-capped Ag NPs (− 46 mV) and the Ag NPs of the mouth spray (− 24 mV) to be negatively charged. No measurements were possible for the Ag NPs of the skin cream.

The composition of surface constituents of the Ag NPs was assessed using Raman spectroscopy (Fig. 2), except for the skin cream that did not give any useful results due to difficulties in finding the Ag NPs in the matrix.

The bare Ag NPs showed the presence of carbonate- and carboxyl-containing compounds as deduced from Raman bands at for example 700, 1131, 1392, 1476, and 1607 cm^−1^ (Fig. 2a) (Kai et al. 1989; McQuillan et al., 1975). Adsorbed silver carbonate has previously been seen to desorb from bare Ag NPs when immersed in solution (Hedberg et al. 2012). A peak at 235 cm^−1^ indicates the presence of Ag-O species (Kai et al. 1989). Observed Raman bands from the Ag NPs in the mouth spray (Fig. 2b) were more difficult to assign but most likely related to components containing C-H (1215, 2931 cm^−1^), C-C (1550, 1586 cm^−1^), C-O (1153, 1215 cm^−1^), and COO^−^ and/or C-N groups (1315, 1365, 1550, 1586 cm^−1^) (Lin-Vien et al. 1991). The Raman spectra of the PVP-capped Ag NPs (Fig. 2c) confirmed the presence of PVP at the surface as judged by Raman bands at 647, 765 cm^−1^ (ring torsion, out of plane), 941 cm^−1^ (CH out of plane bend), and 1362 cm^−1^ (ring breathing mode) (Mdluli et al. 2009).

The total silver content of the cosmetic products followed a concentration of 31.2 ± 0.3 mg Ag/kg product for the mouth spray and 24.1 ± 0.4 mg Ag/kg product for the skin cream. These concentrations are slightly higher compared with the supplier information (30 and 20 mg/kg, respectively). The measured silver contents of the consumer products were in the range of what has previously been reported for different consumer products, whereas the observed primary sizes of the Ag NPs were in the lower range of earlier observations (Khaksar et al. 2019b; Quadros et al. 2013; Tulve et al. 2015).

Since rapid dissolution already during the preparation of stock solutions has been observed for other metal-containing NPs (Pradhan et al. 2016), such information needs to be reported for any dissolution studies of NPs. For the bare Ag NPs of this study, a very small fraction of silver was dissolved (0.001%, measured with AAS after filtration through a 20 nm membrane) after sonication and dilution to the intended particle concentration (2 mg/L) for exposures in synthetic media. Similar quantities (0.001%) of dissolved silver were observed for the PVP-capped Ag NPs (2 mg/L Ag NPs) after dilution into ASW and AS. Higher levels were observed for the Ag NPs in the mouth spray (0.34%) for the same particle loading as for the bare and PVP-capped Ag NPs.

### Dissolution and particle agglomeration kinetics for Ag NPs in skin cream and mouth spray

**Fig 3:**
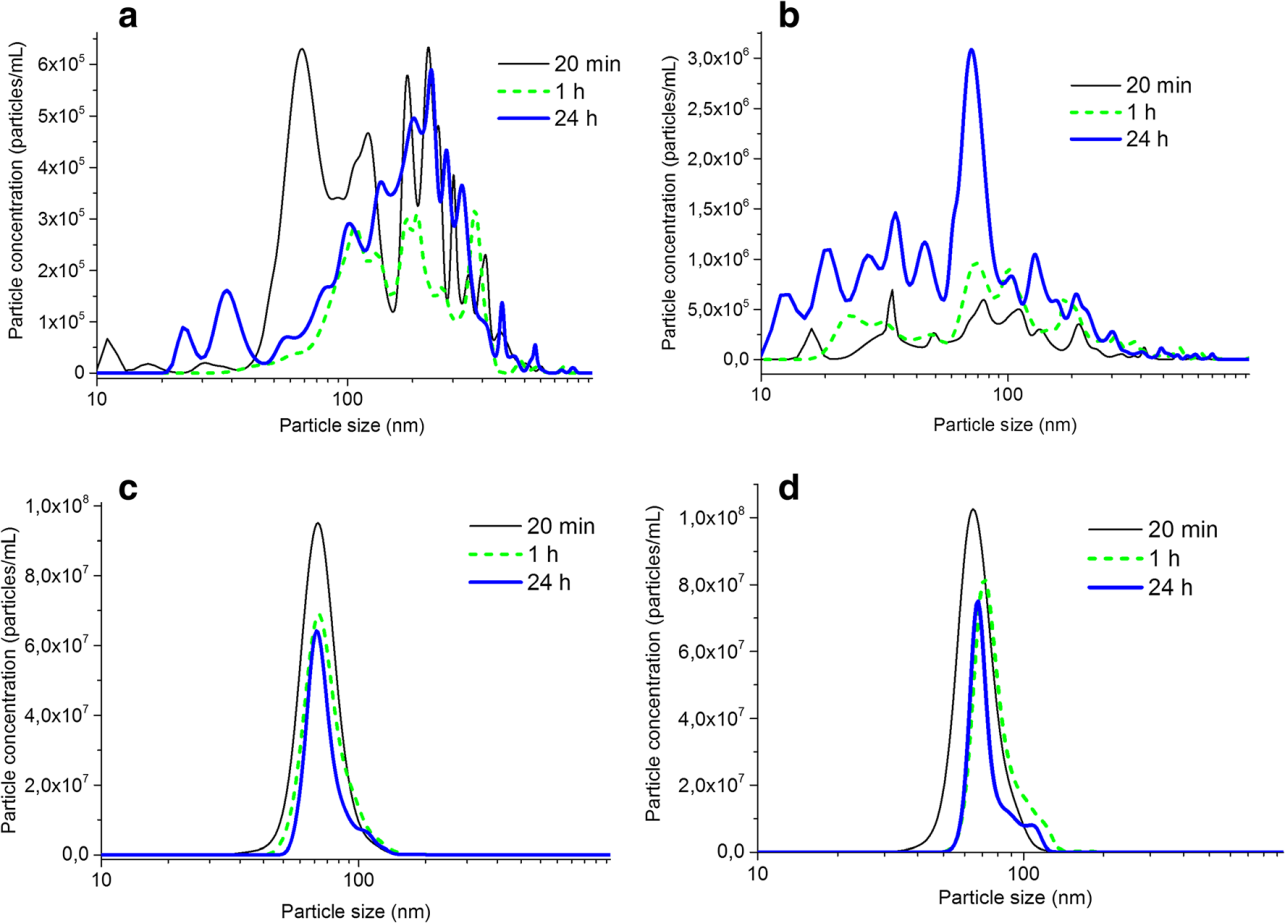
Kinetics of particle size in solution measured by means of NTA, from the Ag NP–containing mouth spray (**a**) and PVP-capped Ag NPs (**c**) in AS, and the Ag NP-containing skin cream (**b**) and PVP-capped Ag NPs (**d**) exposed in ASW. The results display mean results of three independent samples

Particle size kinetics are presented in Fig. 3 for Ag NPs released from the skin cream in ASW (a), mouth spray in AS (b), PVP Ag NPs in ASW (c), and AS (d). In all cases, there was no statistical difference between the different time points due to largely varying results between the replicas.

When compared with the primary sizes of the Ag NPs in mouth spray (5–25 nm, Fig. 1c), agglomeration was evident already within 20 min in AS seen from the polydisperse size distributions with sizes ranging from typically 10 to 800 nm. Similar observations were made for the Ag NPs released from the skin cream (primary sizes 15–25 nm, Fig. 1d) into ASW with a particle size distribution ranging from approximately 15 to 500 nm after 20 min. The extensive agglomeration and sedimentation observed for the bare Ag NPs in both AS and ASW (data not shown) disabled any size-quantification by means of NTA and DLS and imply rapid formation of micron-sized agglomerates. Rapid agglomeration of bare Ag NPs in ASW has been reported earlier (Hedberg et al. 2014b; Pinďáková et al. 2017) and is related to the relatively high ionic strength of ASW in this work (Table 1) which shields electrostatic forces, combined with the relatively high attractive van der Waals forces for metal NPs (Pradhan et al. 2016). The higher colloidal stability of the Ag NPs in the mouth spray and the skin cream compared with the bare Ag NPs can to some extent be explained by the presence of adsorbed species (e.g., organic compounds, and residues from the skin cream formulation; see Fig. 3) that can provide electrostatic and steric effects that improve their colloidal stability.

The PVP Ag NPs showed little agglomeration in ASW and AS due to the stabilizing effect of the PVP capping agent, as the main peak in the size distribution was centered close to their primary size at approximately 67 nm.

**Fig. 4:**
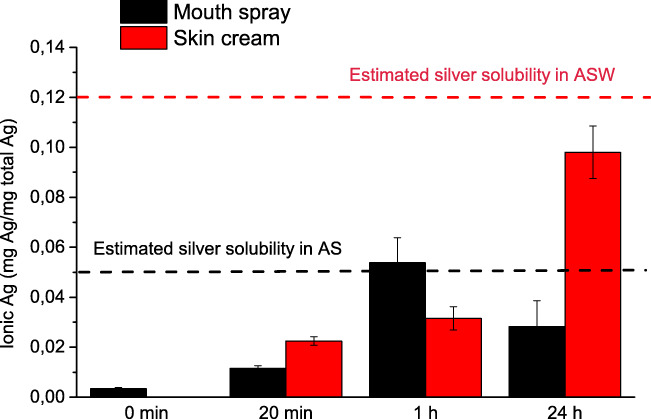
Dissolution kinetics of the Ag NPs of the skin cream in ASW and of the Ag NPs of the mouth spray in AS. The estimated solubility (Medusa software) of silver at equilibrium conditions in ASW and AS is given as the dashed lines. The results display the mean and standard deviation of three independent samples and the error bars correspond to the standard deviation between these samples. The 0 min time point corresponds to the amount of ionic silver determined in the stock solution of the mouth spray. No measurements were possible of the corresponding amount of ionic silver for the skin cream prior to exposure

Figure 4 shows the dissolution kinetics for the Ag NPs of the skin cream in ASW and for the Ag NPs of the mouth spray in AS. The particle loading (2 mg Ag NPs /L) is in the same range as investigated in other studies (Quadros et al. 2013). This particle loading represents a dilution of the content in the consumer products with a factor of approximately 10 for the skin cream and 15 for the mouth spray. The results for mouth spray are intended to have implications on an exposure scenario in which Ag NPs in a spray product release ions and NPs into the saliva, not to mimic conditions of the mouth spray aerosol (Quadros and Marr 2011). The results are compared with the estimated solubility of silver in ASW and AS at equilibrium conditions as calculated using the Medusa software (see Fig. S5 for complete Ag-Cl speciation information).

The results show a fast initial dissolution of the Ag NPs of the mouth spray to levels close to the estimated solubility of silver in AS after 1 h exposure. The dissolved fraction was significantly lower after 24 h compared with 1 h (*p* < 0.05, Student’s *t*-test), indicative of precipitation of AgCl that lowers the concentration of ionic silver in solution over time. These observations highlight the importance of considering the Cl^−^/Ag^+^ ratio when conducting dissolution experiments of Ag NPs at chloride-rich conditions (Levard et al. 2012; Levard et al. 2013b). It should be noted that the presence of chlorides also can enhance the dissolution of Ag NPs, especially at under-saturated conditions (Kent and Vikesland 2011).

Rapid dissolution due to the well-known size effect that result in higher solubility and dissolution rates for particles smaller than 20 nm could also be the case for the Ag NPs (primary size of 2 nm) of the mouth spray in this study (Figs. 1, S2) (Hedberg et al. 2019a; Molleman and Hiemstra 2017). Particle agglomeration has however been shown to reduce this nano-specific effect (Allen et al. 2017; Hedberg et al. 2019a). Agglomeration was evident also in this study (Fig. 3), but since NTA cannot detect particles as small as 2 nm, this opens up for the possibility that the rapid initial dissolution kinetics (Fig. 4) is influenced by these small NPs. The presence of surface compounds on the Ag NPs in the mouth spray prior to immersion in AS (Table 1) will also influence the dissolution kinetics (Radwan et al. 2019).

**Fig. 5:**
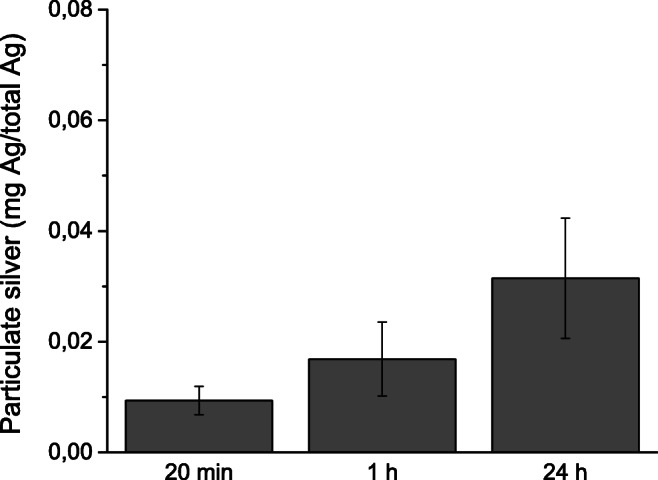
Kinetics of the release of silver as particles (sized > 20 nm) from the Ag NP–containing skin cream exposed in ASW for 20 min, 1 h, and 24 h. The results reflect mean values and standard deviation of three independent samples

The fraction of silver released as particles from the skin cream into ASW is presented in Fig. 5, defined as the fraction of silver retained by the 20-nm pore size membrane (see experimental section). The results show a gradual increase of released silver as particles up to 24 h (3% of the total amount of Ag).

The total amount of released silver (as particles and ions) from the skin cream exposed for 1 h in ASW equals approximately 1 mg Ag/kg product (for a mass to solution ratio of 1:20). This amount is within the range reported by Quadros et al. (0.14–18.5 mg Ag/kg) for a variety of Ag NP–containing products tested in ASW, even though that study was performed using a mass to solution ratio of 1:50 and a time period 2 h (Quadros et al. 2013). Considering that the skin cream only released ca. 3% of its Ag NPs into solution (Fig. 5), the amount of ionic silver in solution was high (10% of the total silver content). As discussed above, the presence of the small-sized primary particles within the skin cream could possibly explain the relatively high level of ionic silver (Hedberg et al. 2019b). Also, it is possible that adsorbed organic matter could reduce the chemical stability of the Ag NPs (Molleman and Hiemstra 2017). Radwan et al. furthermore reported that constituents of the consumer product could influence the dissolution kinetics, although the mechanistic understanding is still unknown (Radwan et al. 2019).

In all, the results clearly show that most of the Ag NPs in the mouth spray and the skin cream would remain as NPs even after 24 h exposure in AS and ASW as less than 10% of the NPs had dissolved within this time frame. It is anticipated that further transformations of the Ag NPs will take place if transported via, for example, the graywater to the wastewater treatment plant (Hedberg et al. 2014a; Kaegi et al. 2011; Khaksar et al. 2019b), by interactions with other ligands such as sulfides (Levard et al. 2011), and as a result in coating degradation over time (Kirschling et al. 2011).

Table 3 summarizes comparisons between the Ag release from products in this work with other investigated products in AS and ASW.

**Table 3:** Comparisons of release of Ag from consumer products containing Ag NPs. The Ag release from this work corresponds to the release after 24 h

Product	Ag NP content in product (mg Ag/kg product)	Ag NP size in product (nm)	Solution for Ag release test	Total Ag release (mg Ag/kg product)	Reference
Skin cream	24.1 ± 0.4	15–25	Artificial saliva	0.3 ± 0.1 (ionic silver release)	This work
Baby blanket	109.8 ± 4.1	30–100	Artificial saliva	1.2 ± 0.1	Quadros et al. 2013) (Tulve et al. 2015)
Plush toy: interior foam	48.2 ± 5.0	20	Artificial saliva	1.77 ± 0.03	Quadros et al. 2013) (Tulve et al. 2015)
Plush toy: interior foam	48.2 ± 5.0	20	Artificial saliva	1.77 ± 0.03	Quadros et al. 2013) (Tulve et al. 2015)
Mouth spray	31.2 ± 0.3	2–20	Artificial sweat	1 ± 0.2	This work
Baby blanket	109.8 ± 4.1	30–100	Artificial sweat	4.8 ± 0.3	(Quadros et al. 2013; Tulve et al. 2015)
Plush toy: interior foam	48.2 ± 5.0	20	Artificial sweat	18.5 ± 1.1	Quadros et al. 2013) (Tulve et al. 2015)
Different fabrics	36.2–425.2	200–500	Artificial sweat	15.5–322	(Kulthong et al. 2010)

There are some differences in the reported compositions of artificial sweat and saliva, which makes the comparisons with literature somewhat preliminary. We can note that one other investigations for Ag NPs in artificial sweat used higher NaCl concentrations (10.8 g/L instead of 5 g/L) (Quadros et al. 2013) which could induce more release of silver compared with this work.

The bare Ag NPs agglomerated more than the NPs of the mouth spray and skin cream, and particle size can largely affect the extent of dissolution (Hedberg et al. 2019b). The next section explores if the geometric surface area is a way forward to compare dissolution data of differently sized NPs of varying characteristics in consumer products.

### Prospects of using geometric surface area to compare dissolution data of NPs in consumer products of varying characteristics

The following discussion is based on using the geometric surface area of the NPs for normalization of dissolution to explore the possibility to improve comparisons and read-across of Ag NP dissolution data. The geometric surface area is here defined as the area calculated from the primary size determined from electron microscopy imaging prior to any exposure (e.g., Fig. 1).

The wide range of primary Ag NP sizes, as previously reported for different consumer products (Benn and Westerhoff 2008; Quadros et al. 2013; Tulve et al. 2015), makes it difficult to select which sizes of bare Ag NPs that could be used as models for Ag NPs in consumer products. Dissolution/transformation data of bare Ag NPs may further not always be a good proxy for the short-term behavior of Ag NPs in consumer products. Acute toxic potency of Ag NPs has, for example, been shown to at least to some extent be connected to both particles and to released amounts of ionic silver (Zhang et al. 2016). The results presented in the following are intended to be used as a starting point for further investigations with the aim to find model systems applicable to assess transformation/dissolution scenarios of Ag NPs in consumer products.

**Fig. 6:**
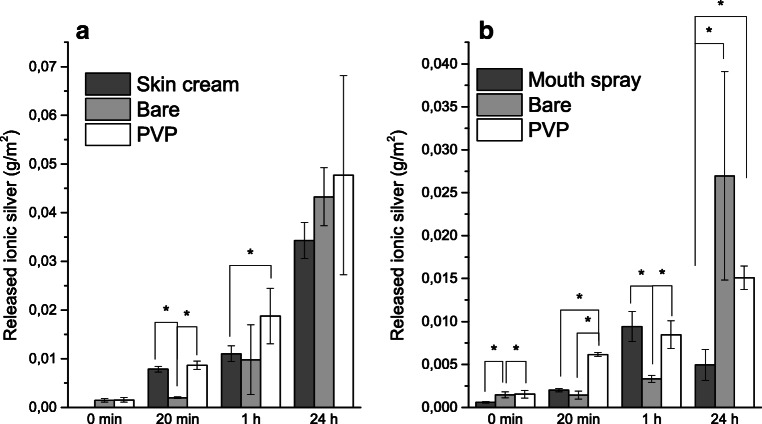
Release of ionic silver from Ag NPs (after 0 min, 20 min, 1 h, and 24 h normalized to the geometric surface area. **a** Ag NPs in skin cream, bare Ag NPs, and PVP-capped Ag NPs in ASW. **b** Ag NPs in mouth spray, bare Ag NPs, and PVP-capped Ag NPs in AS. The stars indicate significant differences, Student’s *t*-test, *p* > 0.05

Figure 6 shows dissolution data normalized to the geometric surface area of the Ag NPs in mouth spray skin cream, PVP-capped Ag NPs, and bare Ag NPs. Normalization with geometric surface area to some extent evens out the dissolution results for the Ag NPs in the skin cream, the bare Ag NPs, and the PVP-capped Ag NPs in ASW for the time periods longer than 1 h. The relatively small differences in the normalized dissolution results imply a greater effect of surface area on the dissolution kinetics compared with, for example, differences in primary size that may lead to faster dissolution for particles sized less than 20 nm (Hedberg et al. 2019b) and effects of adsorbed surface species or capping agents such as in the case of the PVP-capped Ag NPs.

Conversely, normalized dissolution data from the exposure in AS (Fig. 6b) showed large and significant differences for most of the investigated time points. This may be a consequence of released silver levels in solution being close to the saturation level in the case of the Ag NPs from the skin cream (Fig. 4). This saturation can influence the release kinetics (Kuech et al. 2016) and thus make them less comparable to the dissolution of both bare and PVP-capped Ag NPs.

The smaller particle size of the Ag NPs in the consumer products compared with the pristine can in general lead to faster dissolution rates (Kuech et al. 2016). The agglomeration (Fig. 3) will however make this effect smaller and make the properties (e.g., corrosion potentials) similar to larger-sized NPs (Allen et al. 2017).

**Fig. 7:**
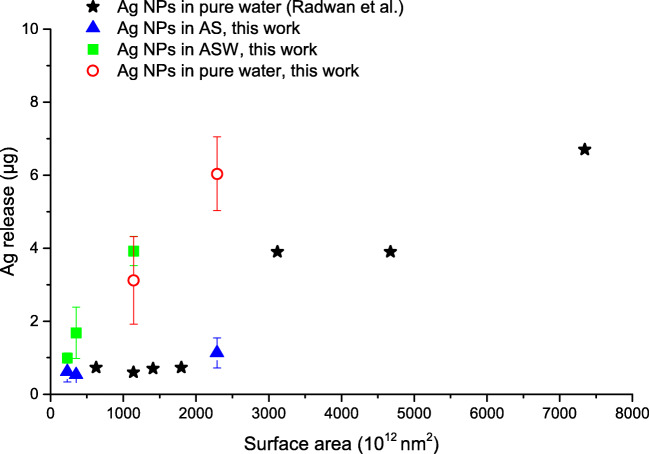
Dissolution (released ionic silver) after 24-h exposure in AS (filled triangles), ASW (filled squares), and pure water (open circles). Data from Radwan et al. is included for different kinds of Ag NPs (stars) (Radwan et al. 2019). The surface area reflects the total geometric surface area of particles in each experiment calculated from electron microscopy imaging of particle sizes prior to exposure

Figure 7 shows the extent of dissolution of Ag NPs vs. geometric surface area after 24 h in pure water, AS, and ASW for different Ag NPs, including results of this study and literature findings (Radwan et al. 2019). The Ag NPs presented in Fig. 7 reflect very different characteristics, and they were exposed in different solutions, all of which will have an impact on dissolution process (Hedberg et al. 2019b). Nonetheless, the results show possibly quasi-linear behaviors (although there are too few data points), similar as the clear trend with increased dissolution with increased surface area evident from the results of Radwan et al. on different Ag NPs (bare and in consumer products) (Radwan et al. 2019).

Based on the results in Fig. 7, there are somewhat promising prospects of using the geometric area for simplifying the comparison of dissolution data of Ag NPs from different consumer products. This simplified approach however comes with a price in terms of a relatively wide range of dissolution rates for a given solution. This uncertainty is in line with the relatively large ranges of dissolution rates observed for Ag NPs in freshwater-like media (Hedberg et al. 2019b; Mitrano et al. 2014). Further investigations need to be conducted in order to assess whether there is a clear trend in terms of surface area versus dissolution for different kinds of solutions. This could pave the way for regressions of dissolution rate versus surface area, data that could be used to support modeling and risk assessments of Ag NPs (Caballero-Guzman and Nowack 2016; Mitrano et al. 2015b; Mitrano and Nowack 2017). An even better area for normalization would be the effective surface area in solution (Dale et al., 2017), which is influenced by, for example, fractal dimensions and porosity that influence the dissolution process (He et al. 2013). However, its applicability is limited as it is much more difficult to assess experimentally than the geometric surface area.

## Conclusions

This study investigated the transformations of Ag NPs in mouth spray and skin cream, in artificial sweat and saliva solutions for up to 24 h. Agglomeration was evident in both solutions for the small-sized primary Ag NPs (< 25 nm) resulting in particle agglomerates sized several hundred nanometers. The dissolution of silver was after 24 h less than 10% (with only a few percent released as NPs). The results show that Ag NPs in these consumer products to a large extent will remain as NPs also when becoming mobile in saliva and/or sweat.

Data was compiled to explore the use of geometric surface area of Ag NPs based on electron microscopy imaging as a way to compare dissolution data from Ag NPs in consumer products with the behavior of bare Ag NPs as model particles. The results indicate that the normalization of dissolution with the geometric surface area is promising a way forward as a method to group the dissolution characteristics of Ag NPs from different consumer products. Further investigations are still required to unambiguously conclude its applicability and further use in risk assessment.

## Electronic supplementary material

ESM 1 (DOCX 2.71 mb)

## Data Availability

The datasets used and/or analyzed during the current study are available from the corresponding author on reasonable request.
