# Supplementary material for: Transformation of silver nanoparticles released from skin cream and mouth spray in artificial sweat and saliva solutions: particle size, dissolution, and surface area
Source: Environ Sci Pollut Res Int. 2020 Oct 23;28(10):12968–79. doi: 10.1007/s11356-020-11241-w (PMC7921047; doi:10.1007/s11356-020-11241-w)
Supplement: Supplementary file 1 — (DOCX 2.71 mb) [file 11356_2020_11241_MOESM1_ESM.docx]

# Supporting information

**Transformation of silver nanoparticles released from skin cream and mouth spray at simulated skin and oral contact: particle size, dissolution, and surface area**

Jonas Hedberg,*^,a^ Madeleine Eriksson,^a^ Amina Kesraoui,^a^ Alexander Norén,^a^ Inger Odnevall Wallinder^a^

^a^ KTH Royal Institute of Technology, School of Engineering Sciences in Chemistry, Biotechnology and Health, Department of Chemistry, Division of Surface and Corrosion Science, Stockholm, Sweden

* corresponding author, e-mail: [jhed@kth.se](mailto:jhed@kth.se)


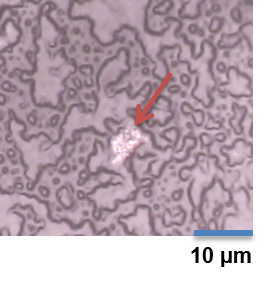


**Figur S1**. Optical microscope image of drop cast mouth spray with the red arrow indicating the location for the Raman spectrum collected in Figure S3.

**
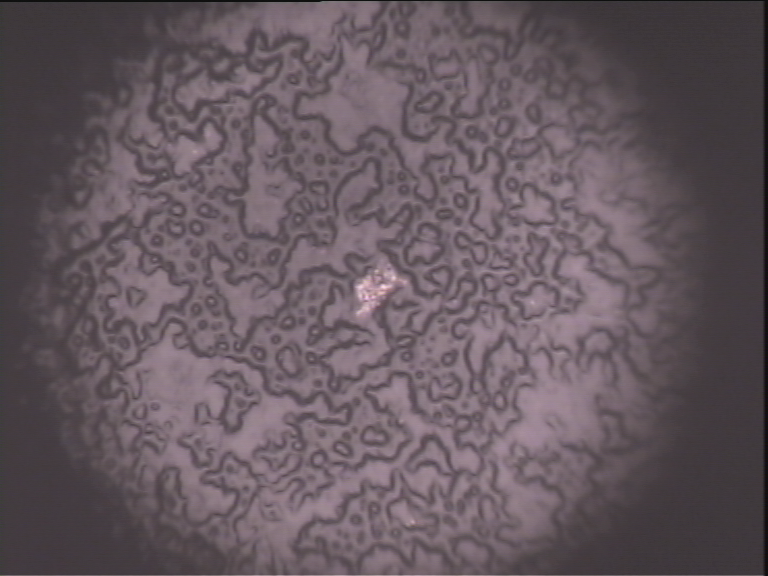
**


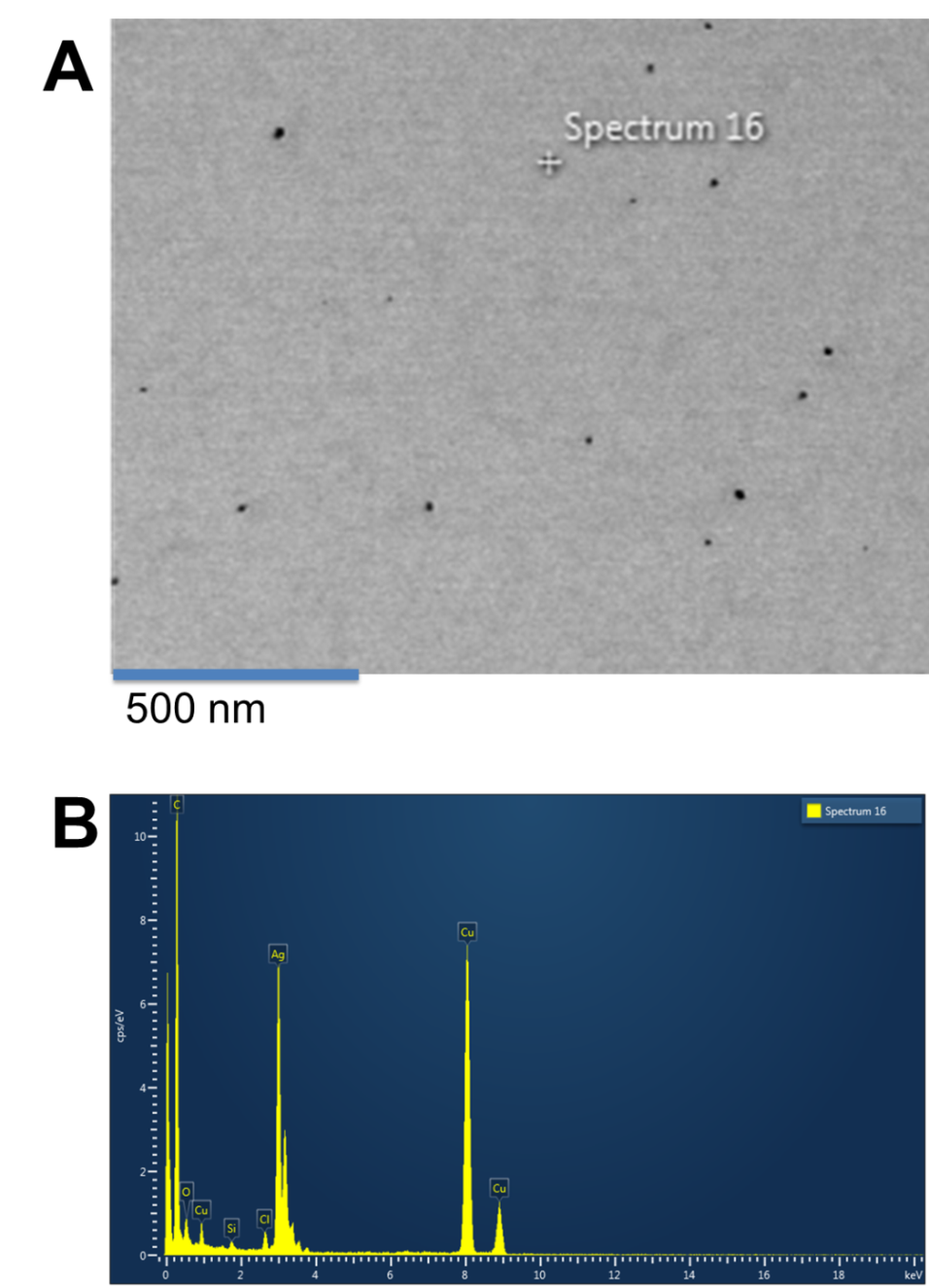


Figure S2. A: TEM image of mouth spray containing Ag NPs deposited on a copper TEM grid. . Their silver content was confirmed using EDS at the spot marked Spectrum 1, showed in B.

##
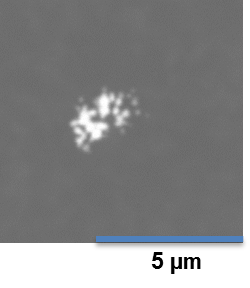


Figur S3. SEM image (backscattered electrons) of drop cast mouth spray containing Ag NPs. The presence of silver in the white areas was confirmed using EDS (data not shown)


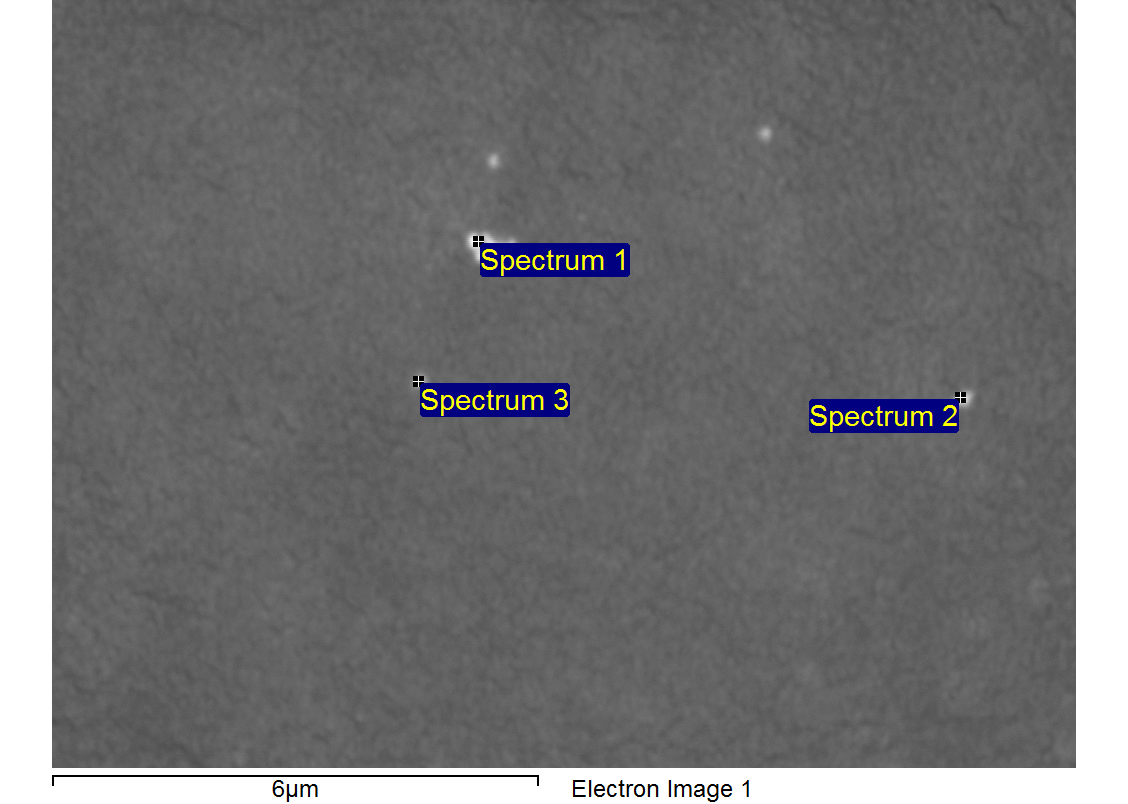


Figure S4. SEM image of Ag NPs in skin cream. A thin gold layer was evaporated on top of a layer of skin cream in order to make the sample conducive for the SEM analysis. The marked spots correspond to positions were EDS spectra were collected. See Table below for resulting wt% of the different element. The sizes of the Ag NPs clusters are ca. 150 nm.

**Table S1**. EDS data from spots investigated in Figure S4

| Spectrum | C | O | Na | Ag | Au | Total |  |
| --- | --- | --- | --- | --- | --- | --- | --- |
|  |  |  |  |  |  |  |  |
| Spectrum 1 | 71.34 | 10.02 | 0.70 | 9.68 | 8.27 | 100.00 |  |
| Spectrum 2 | 76.25 | 9.52 | 0.57 | 5.86 | 7.80 | 100.00 |  |
| Spectrum 3 | 78.63 | 12.62 | 0.34 | 0.00 | 8.40 | 100.00 |  |


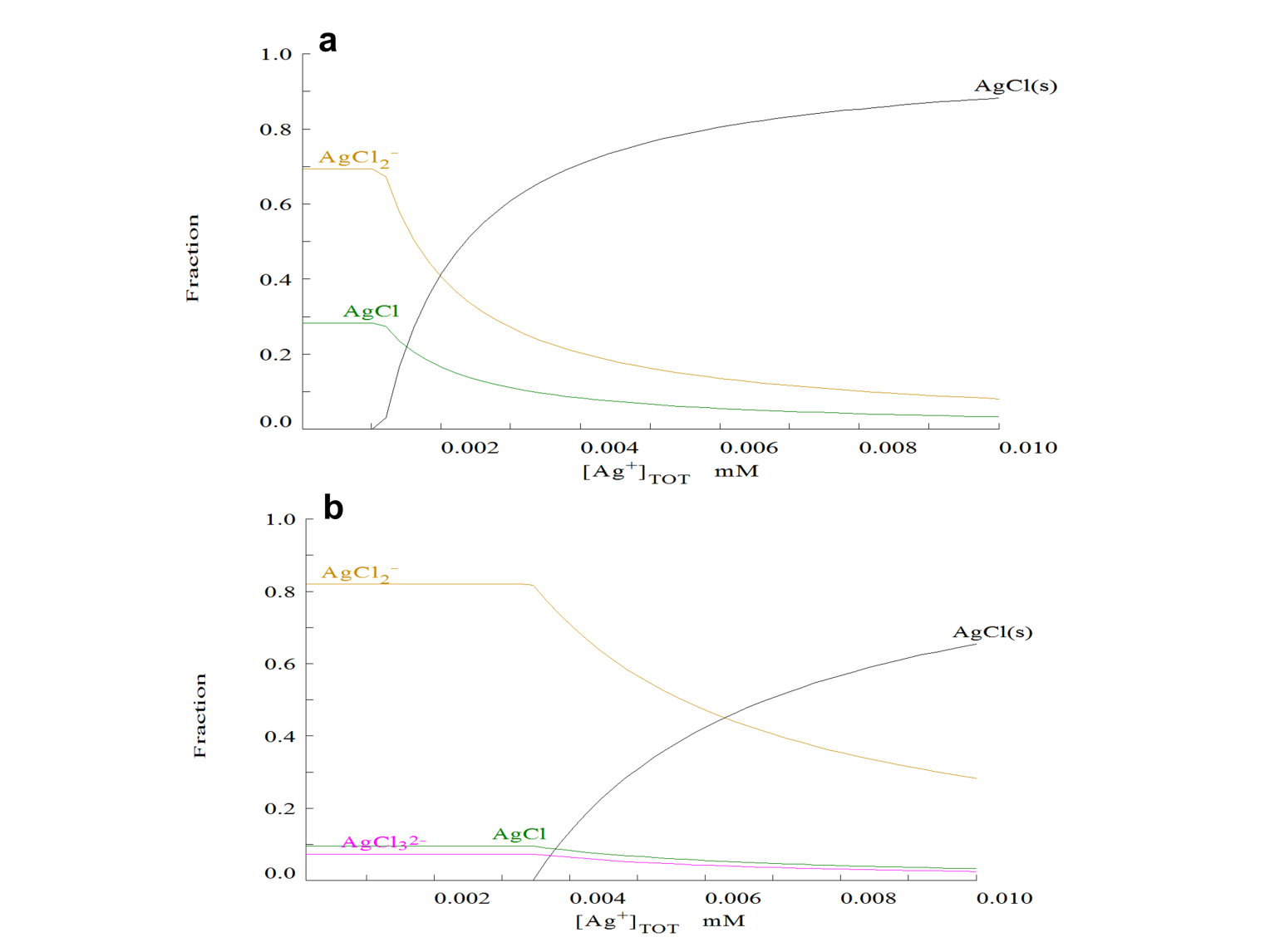


Figure S5. Medusa equilibrium calculations for AS (A) and ASW (B) for different silver calculations. AgCl(s) represents insoluble silver chloride and the rest of the silver complexes are soluble.
